# Brain arteriovenous malformations of the middle cerebral artery region: image characteristics and endovascular treatment based on a new classification system

**DOI:** 10.1186/s12883-023-03084-y

**Published:** 2023-01-25

**Authors:** Han Su, Jinlu Yu

**Affiliations:** Department of Neurosurgery, First Hospital of Jilin University, 1 Xinmin Avenue, Changchun, 130021 China

**Keywords:** Brain arteriovenous malformations, Middle cerebral artery, Classification, Endovascular treatment, Prognosis

## Abstract

**Background:**

To date, few studies have investigated the use of endovascular treatment (EVT) for brain arteriovenous malformations (BAVMs) in the supplying area of the middle cerebral artery (MCA). Moreover, no suitable classification was aimed at EVT for MCA-BAVMs. Therefore, this study proposed a new classification.

**Methods:**

This study retrospectively collected 135 MCA‑BAVMs. They were classified into four types: Type I BAVMs located above the M1 segment; Type II BAVMs located in the region around the Sylvian fissure; and Type III BAVMs located in the supplying region of the M4 segment and subdivided into types IIIa and IIIb. The relevance of various types of MCA-BAVMs and their imaging characteristics and EVT outcomes was analyzed by ordinary one-way ANOVA, Tukey's multiple comparisons test and the chi-square test.

**Results:**

The 135 patients averaged 33.8 ± 14.7 years and included 75 females (55.6%, 75/135). Among them, 15 (11.1%, 15/135), 16 (11.9%, 16/135), 54 (40%, 54/135), and 50 (37%, 50/135) MCA-BAVMs were type I, II, IIIa and IIIb, respectively. After EVT, a good outcome was achieved in 97% of patients. Statistical analysis showed that type I BAVMs were smaller than type II and IIIb BAVMs (*P* value < 0.05), and type IIIb BAVMs were larger than type I and IIIa BAVMs (*P* value < 0.05). Deep vein involvement in type I and IIIb BAVMs was more common than in other types (*P* value < 0.05), and intraventricular hemorrhage (IVH) was also more common (*P* value < 0.05). The normal morphology in type IIIb was less than that in the other types (*P* value < 0.05). Type IIIa BAVMs had a higher degree than other types (*P* value < 0.05).

**Conclusion:**

The present study demonstrated that the new classification of MCA-BAVMs can be used to evaluate imaging characteristics and EVT outcomes in different types. In addition, EVT may be a safe treatment modality for MCA‑BAVMs.

## Background

A brain arteriovenous malformation (BAVM) is a congenital vascular disease with an abnormal nidus that occurs between arteries and veins and lacks an intervening capillary network [1]. BAVMs can occur in the supplying areas of different intracranial arteries, such as the anterior cerebral artery (ACA), posterior cerebral artery (PCA), and vertebrobasilar artery [2–4]. BAVMs can also occur in the supplying area of the middle cerebral artery (MCA), termed MCA‑BAVMs, and they are unique due to the complex anatomy of the MCA [5].

The MCA can be divided into four segments: M1 (sphenoidal), M2 (insular), M3 (opercular), and M4 (cortical) [6, 7]. Therefore, MCA‑BAVMs had a wide distribution along the MCA course, and an appropriate classification was necessary to guide the treatment. Currently, treatment options for BAVMs include endovascular treatment (EVT), surgical resection, radiotherapy, and medical therapy [8]. Of these options, EVT has an important role in the curative embolization of low-grade BAVMs or the adjunctive embolization of high-grade BAVMs in reducing the risk of surgery and radiosurgery [9–11].

However, how can we select cases and guide EVT for MCA-BAVMs? There is no standard grading scheme. Therefore, in this study, we conducted a retrospective single-center investigation of MCA-BAVMs that accepted EVT, and a classification of MCA-BAVMs was proposed to evaluate EVT. At the same time, the imaging characteristics of MCA-BAVMs and the safety of EVT for treating this disease were studied.

## Materials and methods

Patients with BAVMs supplied only or primarily by the MCA system treated by EVT from June 2015 to January 2021 were consecutively enrolled. This study was approved by the institutional ethics committee (No. 2022–405). All methods were performed in accordance with the relevant guidelines and regulations.

### Inclusion and exclusion criteria

Inclusion criteria were as follows: (a) BAVMs mainly located in the supplying region of the MCA, including the M1-4 segments; (b) these BAVMs had only or primary (if not the only) arterial supply from the MCA; and (c) EVT was performed via only the MCA or via both the MCA and other feeding arteries. The exclusion criteria were as follows: (a) MCA-BAVMs with previous EVT, open surgery, or radiosurgery and (b) MCA-BAVMs embolized by feeding arteries other than the MCA.

### MCA-BAVM classification

**Fig. 1:**
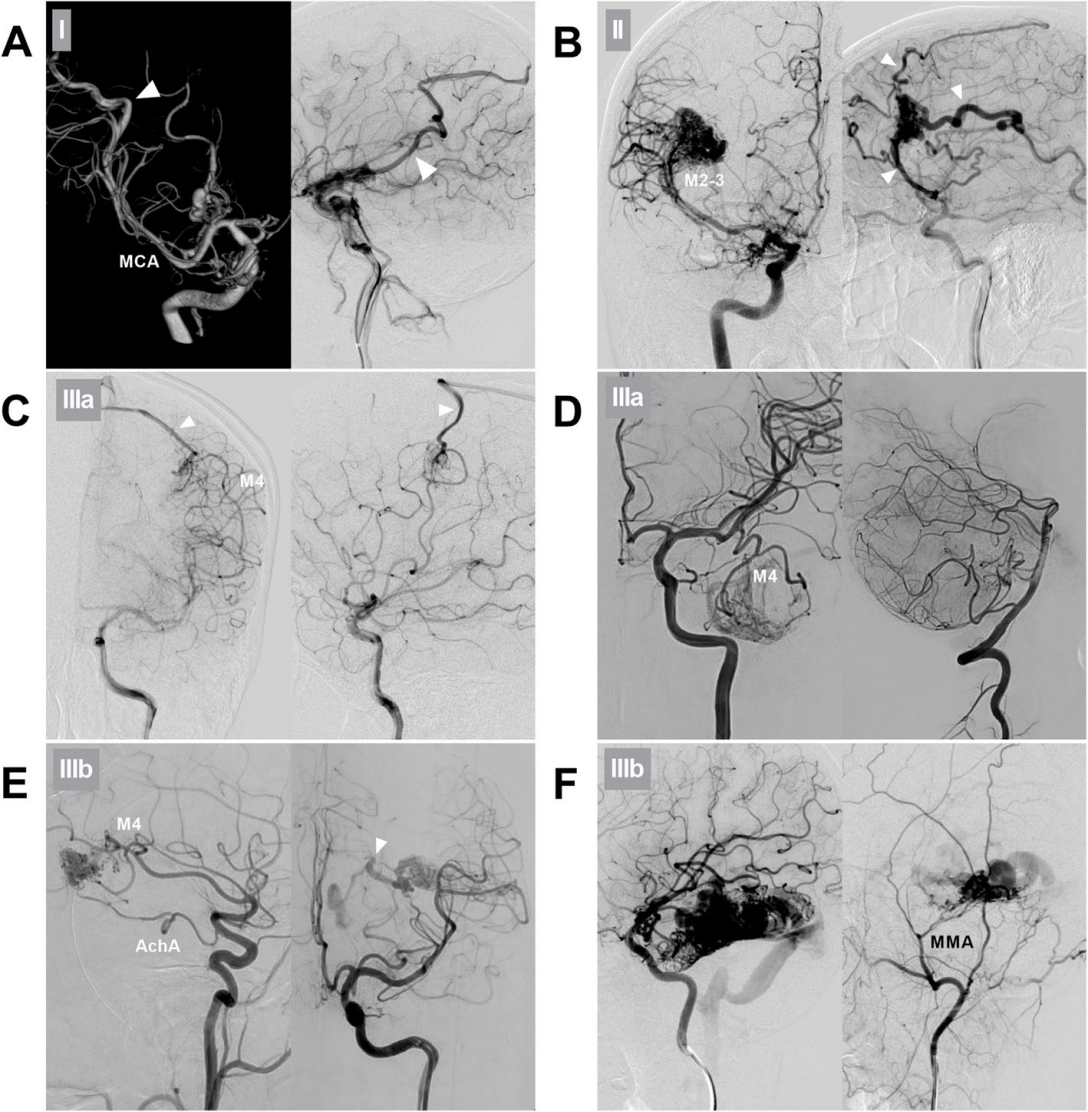
Classification of MCA-BAVMs. **A** Type I BAVM. Three-dimensional angiogram (left) and two-dimensional angiogram (right) of the ICA show that the BAVM above the MCA trunk is supplied by the LSA of the M1 segment. The arrowhead indicates the superficial draining vein. **B** Type II BAVM. Arterial phase (left) and late arterial phase (right) angiograms of the ICA showing the BAVM in the Sylvian fissure supplied by the branches of the M2-3 segments. Arrowheads indicate multiple superficial draining veins. **C** Type IIIa BAVM. ICA angiograms of anteroposterior view (left) and lateral view (right) show that the BAVM in the parietal lobe supplied only by the termination of the M4 segment. The arrowhead indicates a superficial draining vein. **D** Type IIIa BAVM. ICA angiogram (left) and vertebral artery angiogram (right) show that the BAVM in the temporal lobe is supplied only by the temporal branch of the M4 segment. **E** Type IIIb BAVM. Lateral view (left) and anteroposterior view (right) of the ICA angiograms show that the BAVM is supplied by both the AchA and M4 segment. The arrowhead indicates the deep draining vein. **F** Type IIIb BAVM. ICA angiogram (left) and external carotid artery (right) angiogram show that the BAVM is supplied by the MCA and MMA. Abbreviations: AchA, anterior choroidal artery, BAVM: brain arteriovenous malformation, ICA: internal carotid artery, LSA: lenticulostriate artery, MCA: middle cerebral artery, M1-4: first-fourth segments of  the MCA, MMA: middle meningeal artery

MCA-BAVMs were classified into four types according to their positional relationship with the M1-4 segments. Type I BAVMs are located above the M1 segment and are mainly fed by the lenticulostriate artery (LSA); occasionally, branches from the A1 segment of the ACA or the M2 segment can be involved (Fig. 1A). Type II BAVMs are located in the region around the Sylvian fissure and are mainly fed by the branches of the M2 and M3 segments; occasionally, the LSA and the branch of the M4 segment can be involved (Fig. 1B). Type III BAVMs were located in the supplying region of the M4 segment and subdivided into IIIa BAVMs (only supplied by the MCA) (Fig. 1C-D) and IIIb BAVMs (supplied by the MCA and other feeders) (Fig. 1E-F).

### Preoperative data collection

**Fig. 2:**
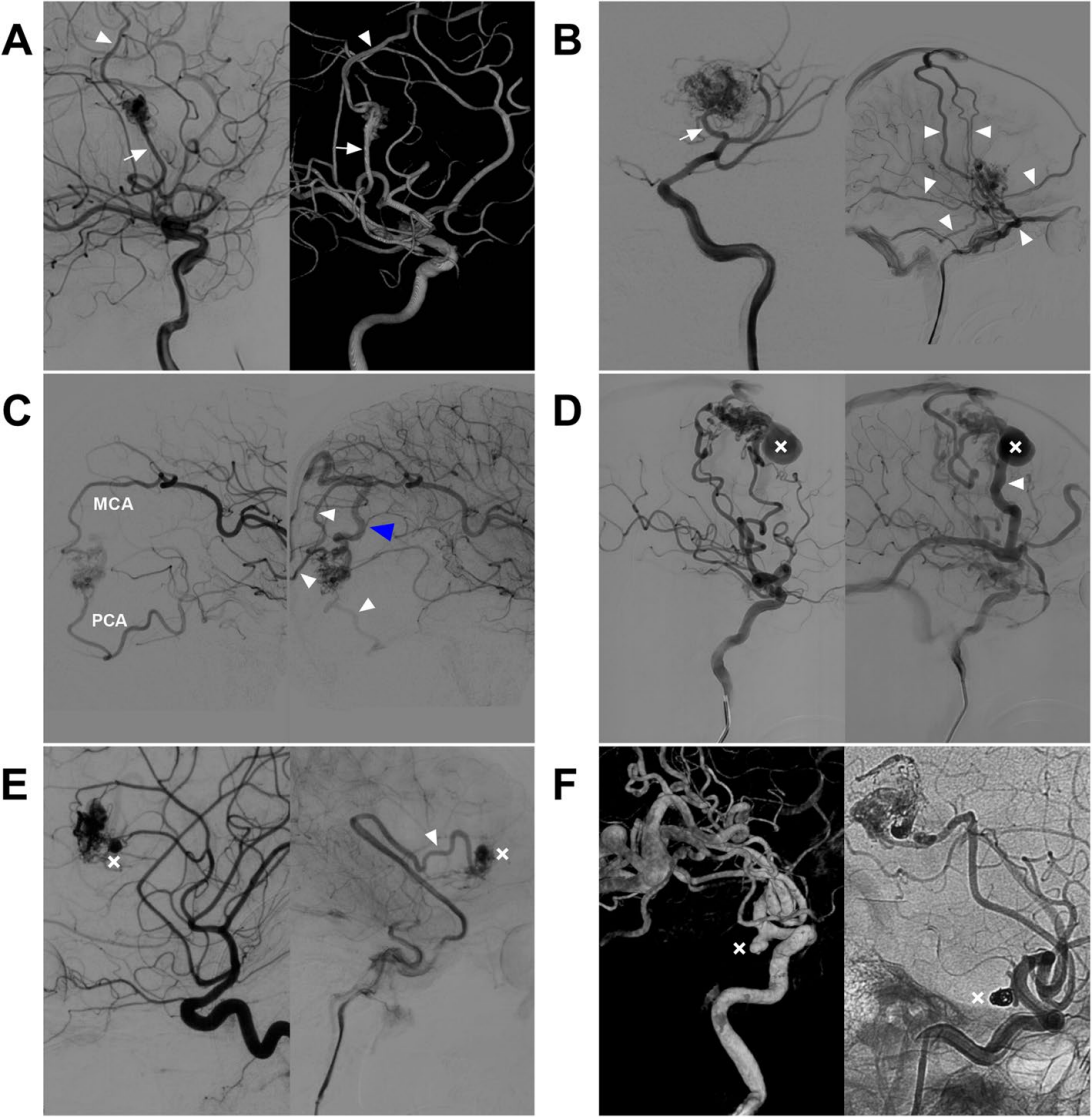
Angiographic characteristics of MCA-BAVMs. **A** Two-dimensional angiogram (left) and three-dimensional angiogram (right) of the ICA show that the BAVM with a simple architecture was supplied only by the MCA (arrow); the only draining vein (arrowhead) is normal, without dilation. **B** The arterial phase angiogram (left) and venous phase angiogram (right) of the ICA show that the BAVM is supplied only by the MCA (arrow), but it has multiple draining veins (arrowheads), and almost all superficial veins of the hemisphere surface are involved. **C** Arterial phase angiogram (left) and late arterial phase angiogram (right) of the ICA show that the BAVM is supplied by the MCA and PCA, it has multiple draining veins (arrowheads), and the primary vein (blue arrowhead) is dilated. **D** Arterial phase angiogram (left) and late arterial phase angiogram (right) of the ICA show a venous aneurysm (forks) on the primary varicose superficial draining vein (arrowhead). **E** Arterial phase angiogram (left) and venous phase angiogram (right) of the ICA show an intranidal aneurysm (fork in left image) in the BAVM; the arrowhead in right image indicates a superficial draining vein. **F** Three-dimensional angiogram of the ICA (left) showing that the BAVM is supplied by the MCA, and an aneurysm (arrow) can be seen on the posterior communicating artery. The aneurysm was coiled (right), and the BAVM was partially embolized. Abbreviations: BAVM: brain arteriovenous malformation, ICA: internal carotid artery, MCA: middle cerebral artery, PCA: posterior cerebral artery

Preoperative data collection included age and sex, clinical presentation, and Hunt-Hess (HH) grade. The HH grade was defined as 0 in unruptured BAVMs. Angiographic characteristics of MCA-BAVMs include the location, feeding artery, nidus size, Spetzler‒Martin (SM) grade, associated aneurysm, draining vein pattern (superficial/deep and single/multiple) and morphology (normal, dilated or varicose) of the primary draining vein (the largest vein that collects most of the outflow from the BAVM (16)) (Fig. 2).

### EVT scheme and strategy

**Fig. 3:**
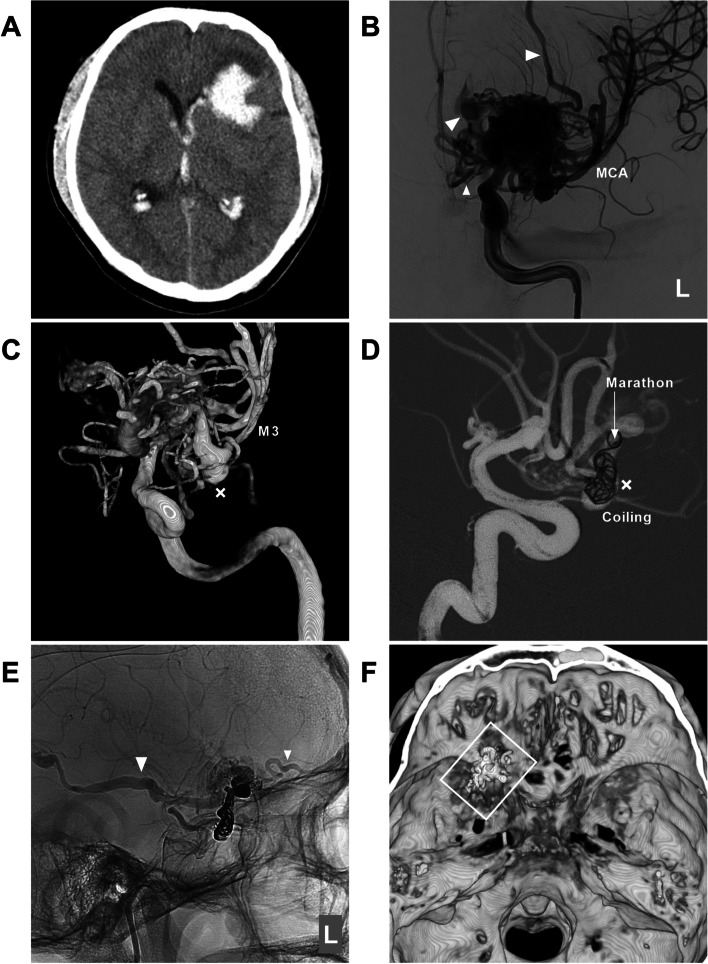
EVT assisted by the pressure cooker technique. **A** CT shows frontal hemorrhage above the Sylvian fissure. B-C: Two-dimensional angiogram (**B**) and three-dimensional reconstruction angiogram (**C**) of the left ICA show that the BAVM is supplied by the M3 segment multiple draining veins (arrowheads), and an aneurysm (fork in C) on the feeding artery. **D** Road-map navigation angiogram showing that the aneurysm (fork) has been coiled to create a pressure cooker effect; the tip of the Marathon microcatheter (arrow) is advanced beyond the coils. **E** Unsubtracted angiogram of the left ICA showing that the volume of the BAVM nidus is reduced, and the draining veins (arrowheads) are patent. **E** Reconstructive Xper CT showing the location of the Onyx (frame). Abbreviations: BAVM: brain arteriovenous malformation, CT: computed tomography, CTA: computed tomography angiography, EVT: endovascular treatment, ICA: internal carotid artery, L: left, MCA: middle cerebral artery, M3: third segment of the MCA

All patients were treated via a transfemoral approach under general anesthesia. A Marathon or Apollo microcatheter (Medtronic, Irvine, California, USA) was used to access the nidus as close as possible to achieve the wedge position via the MCA. Then, an Onyx liquid embolic system (Medtronic, Irvine, California, USA) was cast. If further EVT was needed, another feeding artery could be chosen to repeat the Onyx casting. In EVT of MCA-BAVMs, the pressure cooker technique can be used [12]. First, a microcatheter for casting Onyx is placed in the wedge position in the MCA, and then a microcatheter for coiling is placed behind the microcatheter for casting Onyx. Before casting Onyx, the feeding artery is coiled to produce the effect of a pressure cooker and avoid reflux during Onyx casting (Fig. 3).

**Fig. 4:**
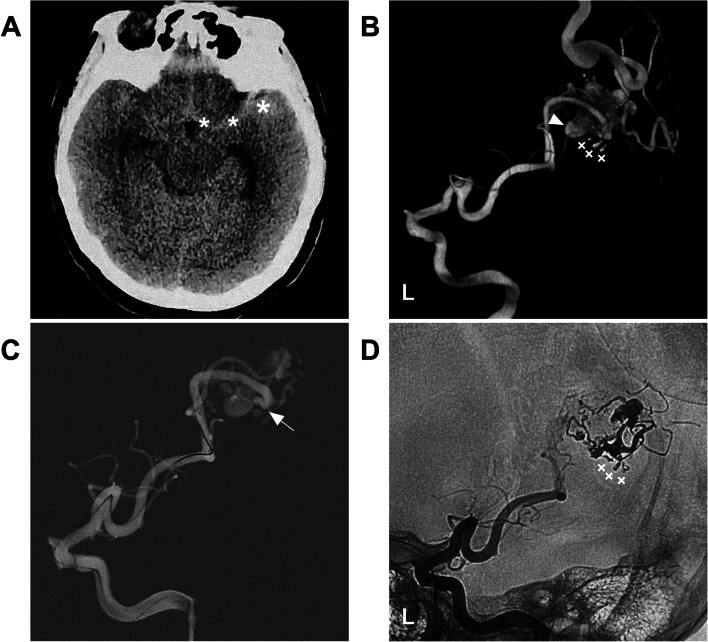
EVT targeting weak structures of BAVMs. **A** CT shows subarachnoid hemorrhage (asterisks) in the left Sylvian fissure. **B** Three-dimensional reconstruction angiogram of the left ICA shows that the BAVM is supplied by the M3 segment with aneurysmal structures (forks) and a venous dilation (arrowhead). **C** Roadmap navigation angiogram shows the microcatheter (arrow) accessed to the BAVM. **D** Unsubtracted angiogram of the left ICA showing Onyx casting in the aneurysmal structures (forks). Abbreviations: BAVM: brain arteriovenous malformation, CT: computed tomography, EVT: endovascular treatment, ICA: internal carotid artery, MCA: middle cerebral artery, M3: third segment of the MCA

**Fig. 5:**
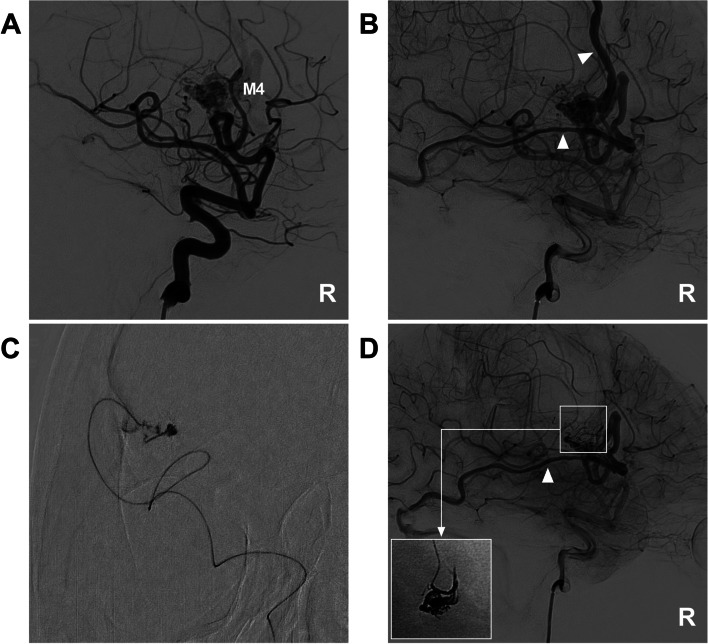
Protection of the draining vein during EVT. **A**-**B** Arterial phase angiogram (**A**) and late arterial phase angiogram (**B**) of the right ICA show that the BAVM is supplied by the M4 segment, and the BAVM has multiple superficial draining veins (arrowheads). **C** Superselective angiogram of the microcatheter showing the microcatheter obtained the wedged position. **D** Right ICA angiogram shows that the primary draining vein is occluded after Onyx casting, and the secondary vein is patent (arrowhead); the picture in the picture shows the casting Onyx. Abbreviations: BAVM: brain arteriovenous malformation, EVT: endovascular treatment, ICA: internal carotid artery, MCA: middle cerebral artery, M4: fourth segment of the MCA, R: right

EVT preferentially targeted dangerous structures, such as associated aneurysms on the feeding artery or dilated structures in the nidus (Fig. 4). If no weak structure is identified, the main purpose of EVT is to reduce the blood flow of BAVMs to help reduce nidus/perinidal angiogenesis [13]. Flow-related aneurysms on a feeding artery away from the nidus could be treated by coiling embolization (Fig. 3D); when those aneurysms were close to the nidus, casting Onyx could be applied (Fig. 4D) [14]. For intranidal aneurysms, the compartment of the nidus containing the aneurysm can be embolized with Onyx casting [15]. In addition, during casting Onyx, it is important to preserve the primary draining vein to prevent intraoperative or acute postoperative hemorrhagic complications due to venous hypertension (Fig. 5) [16, 17].

### Prognosis evaluation

EVT outcomes and complications, length of hospital stay, and Glasgow Outcome Scale (GOS) score at discharge were all recorded. A good outcome was defined as GOS scores of 4 and 5.

### Statistical analysis

GraphPad software (LLC, San Diego, USA) was used for statistical analysis. Continuous variables are expressed as the mean ± standard deviation, and ordinary one-way ANOVA and Tukey's multiple comparisons test were used. The chi-square test was used to compare count data. A *P value* < 0.05 was considered to indicate a significant difference.

## Results

### General information

One hundred thirty-five patients with MCA-BAVMs were enrolled, aged 8 to 68 years (mean, 33.8 ± 14.7 years) and included 75 females (55.6%, 75/135). Among 135 BAVMs, 40 had unruptured BAVMs (29.6%, 40/135), and 95 had ruptured BAVMs (70.4%, 95/135) presenting with various types of intracranial hemorrhages. Among patients with ruptured BAVMs, HH grades I and II were common. The detailed data are shown in Table 1.

**Table 1:** Clinical data in the present study

Characteristic	Data	
Age (years)	Mean	33.8 ± 14.7
	Range	8–68
Sex	Female	55.6% (75/135)
	Male	44.4% (60/135)
Presentation	Unruptured	29.6% (40/135)
	IH	50.4% (68/135)
	IVH	9.6% (13/135)
	IH and IVH	8.1% (11/135)
	SAH	2.2% (3/135)
Hunt-Hess grade	0	29.6% (40/135)
	I	29.6% (40/135)
	II	27.4% (37/135)
	III	13.3% (18/135)
BAVM size (cm)	Mean	3.7 ± 1.7
	Range	0.5–8
Spetzler‒Martin grade	I	20.7% (28/135)
	II	33.3% (45/135)
	III	37% (50/135)
	IV	8.1% (11/135)
	V	0.7% (1/135)
BAVM type	I	11.1% (15/135)
	II	11.9% (16/135)
	IIIa	40% (54/135)
	IIIb	37% (50/135)
Associated aneurysm	Prenidal	5.2% (7/135)
	Intranidal	9.6% (13/135)
Draining vein type	Single superficial vein	47.4% (64/135)
	Multiple superficial veins	40% (54/135)
	Deep vein	8.1% (11/135)
	Superficial and deep veins	4.4% (6/135)
Primary draining vein morphology	Normal	32.6% (44/135)
	Dilated	21.5% (29/135)
	Varicose	45.9% (62/135)
EVT degree of the nidus	< 1/3	16.3% (22/135)
	1/3–2/3	47.4% (64/135)
	> 2/3	36.3% (49/135)
Hospital stay after EVT (days)	Mean	6.7 ± 2.6
	Range	2–13
Glasgow Outcome Scale score at discharge	5	76.3% (103/135)
	4	20.7% (28/135)
	3	3.0% (4/135)

*BAVM* brain arteriovenous malformation, *EVT* endovascular treatment, *IH* intracerebral hemorrhage, *IVH* intraventricular hemorrhage, *SAH* subarachnoid hemorrhage

### Imaging characteristics

Type I BAVMs were found in 15 patients (11.1%, 15/135), Type II BAVMs in 16 patients (11.9%, 16/135), Type IIIa BAVMs in 54 patients (40%, 54/135), and Type IIIb BAVMs in 50 patients (37%, 50/135). The average diameter of the nidus was 3.7 ± 1.7 cm (0.5–8 cm). SM grades I-III were common. Twenty BAVMs had associated aneurysms, including 7 (5.2%, 7/135) aneurysms on the feeding artery and 13 (9.6%, 13/135) intranidal aneurysms. Regarding the pattern of the draining veins, single or multiple superficial veins were common. The morphology of the primary draining vein was dilated in 21.5% (29/135) of BAVMs and varicose in 45.9% (62/135) of BAVMs. The detailed data are shown in Table 1. Regarding the locations of the BAVMs, the temporal lobe was the most common location, accounting for 28.1% (38/135) of locations (Table 2). The feeding arteries of MCA-BAVMs are complex and are shown in Table 3.

**Table 2:** Locations of MCA-BAVMs

Location	Rate
Temporal lobe	28.1% (38/135)
Parietal lobe	19.3% (26/135)
Frontal lobe	18.5% (25/135)
Basal ganglia and insular lobe	9.6% (13/135)
Temporal and parietal lobes	8.1% (11/135)
Lateral ventricular wall	5.2% (7/135)
Sylvian fissure	3.7% (5/135)
Occipital lobe	3.7% (5/135)
Frontal and parietal lobes	1.5% (2/135)
Temporal and occipital lobes	1.5% (2/135)
Parietal and occipital lobes	0.7% (1/135)

*BAVM* brain arteriovenous malformation, *MCA* middle cerebral artery

**Table 3:** Distribution of the feeding arteries of MCA-BAVMs

Feeding artery	Rate	BAVM classification
LSA	9.6% (13/135)	Type I
LSA + A1	0.7% (1/135)	
LSA + M2	1.5% (2/135)	Type I and II
M2 + M3	4.4% (6/135)	Type II
M3	3% (4/135)	
M3 + LSA	0.7% (1/135)	
M3 + M4	5.9% (8/135)	Type II and IIIa
M4	37% (50/135)	Type IIIa
M4 + M3 + MMA	0.7% (1/135)	Type IIIb
M4 + M3 + PCA + MMA	1.5% (2/135)	
M4 + AchA	1.5% (2/135)	
M4 + ACA	5.2% (7/135)	
M4 + ACA + LSA	4.4% (6/135)	
M4 + ACA + LSA + PCA	0.7% (1/135)	
M4 + ACA + MMA	2.2% (3/135)	
M4 + ACA + PCA	1.5% (2/135)	
M4 + MMA	0.7% (1/135)	
M4 + PCA	17% (23/135)	
M4 + PCA + MMA	1.5% (2/135)	

*ACA* anterior cerebral artery, *AchA* anterior choroidal artery, *A1* first segment of the ACA, *BAVM* brain arteriovenous malformation, *LSA* lenticulostriate artery, *MCA* middle cerebral artery, *M1-4* first-fourth segments of the MCA, *MMA* middle meningeal artery, *PCA* posterior cerebral artery

### EVT results and postoperative management

After EVT, 16.3% (22/135) of BAVMs were embolized with a nidus degree of < 1/3, 47.4% (64/135) BAVMs with a degree between 1/3–2/3, and 36.3% (49/135) BAVMs with a degree of > 2/3. Twenty associated aneurysms in MCA-BAVMs were embolized. During EVT, 4 (3.0%, 4/135) patients experienced the complication of intraoperative bleeding; 2 were treated with conservative treatment, and 2 were treated with craniotomy. Overall, hematoma evacuation was performed in 12 patients, and burr hole drainage was performed in 17 patients.

### Prognosis at discharge

The length of hospital stay ranged from 2 to 13 days (6.7 ± 2.6 days). At discharge, a good outcome with GOS scores of 4 and 5 was observed in 97% (131/135) of patients, and 3% (4/135) of patients had a GOS score of 3.

### Statistical analysis

Regarding age and sex, ordinary one-way ANOVA showed a *P* value > 0.05, indicating no significant difference among the four types of BAVMs. For sex, the chi-square test showed a *P* value > 0.05, indicating no significant difference among the four types. The results of the statistical analysis are summarized in Table 4.

**Table 4:** Statistical analysis among classifications

	**Type** I	**Type** II	**Type** IIIa	**Type** IIIb	***P* value**
Age (years)	33.5 ± 9.4	34.3 ± 18.0	33.1 ± 15.8	34.4 ± 14.1	0.9741
Female/total	12/15	8/16	31/54	24/50	0.1674
IVH involvement/hemorrhage	8/14	1/11	3/43	12/27	< 0.0001
Size (cm)	2.3 ± 1.4	3.9 ± 1.4	3.1 ± 1.6	4.7 ± 1.5	< 0.0001
Deep vein involvement/total	11/15	1/16	0/54	5/50	< 0.0001
Normal draining vein/total	5/15	7/16	27/54	5/50	0.0002
EVT degree > (2/3)/total	3/15	4/16	32/54	10/50	0.0001

*BAVM* brain arteriovenous malformation, *EVT* endovascular treatment, *IVH* intraventricular hemorrhage

For age and BAVM size, ordinary one-way ANOVA was used. For other data, the chi-square test was used

Regarding BAVM size, ordinary one-way ANOVA among the four types showed a *P* value < 0.05, Tukey's multiple comparisons test showed that type I BAVMs were smaller than type II and type IIIb BAVMs (*P* value < 0.05), and type IIIb BAVMs were larger than type I and IIIa BAVMs (*P* value < 0.05). Regarding intraventricular hemorrhage (IVH) and deep vein involvement, the chi-square test showed that deep vein involvement in type I and IIIb BAVMs was more common than in other types (*P* value < 0.05), and IVH was more common (*P* value < 0.05). Regarding the morphology of the primary draining vein, the chi-square test showed that the normal morphology in type IIIb was less than that in the other types (*P* value < 0.05). Regarding the EVT degree, the chi-square test showed that Type IIIa BAVMs had a higher degree than other types (*P* value < 0.05). The detailed results of the statistical analysis are summarized in Table 5.

**Table 5:** Multiple comparisons of the data

	***P* value**				
	**IVH involvement/hemorrhage**	**Size**	**Deep vein involvement/total**	**Normal draining vein/total**	**EVT degree > (2/3)/total**
**Type I vs. Type II**	0.0130	0.0234	0.0001	0.5518	0.7393
**Type I vs. Type IIIa**	< 0.0001	0.2785	< 0.0001	0.2522	0.0071
**Type I vs. Type IIIb**	0.4405	< 0.0001	< 0.0001	0.0280	> 0.9999
**Type II vs. Type IIIa**	0.8112	0.2785	0.0643	0.6604	0.0160
**Type II vs. Type IIIb**	0.0372	0.3007	0.6497	0.0023	0.6702
**Type IIIa vs. Type IIIb**	0.0002	< 0.0001	0.0172	< 0.0001	< 0.0001

*EVT* endovascular treatment, *IVH* intraventricular hemorrhage

For size, Tukey's multiple comparisons test was used. For other data, the chi-square test was used

**Fig. 6:**
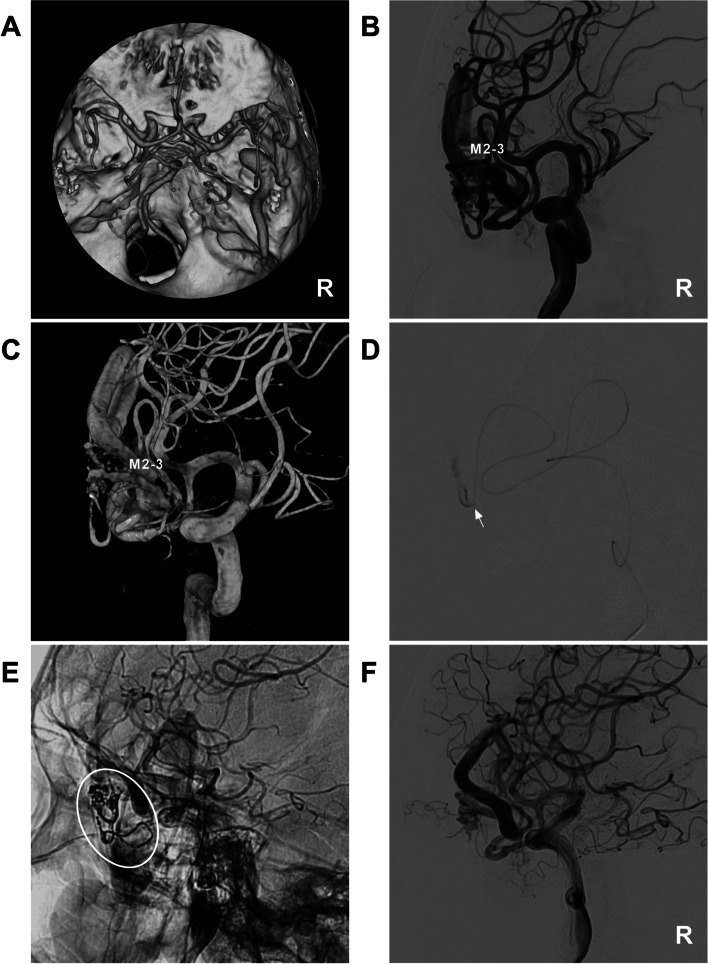
EVT of a type II BAVM. **A** CTA shows a BAVM located in the right Sylvian fissure, and the draining vein is varicose. **B**-**C** Two-dimensional angiogram (**B**) and three-dimensional reconstruction angiogram of the right ICA (**C**) show that the BAVM is supplied by the branches of the M2-3 segments, and the draining vein is varicose. **D** Superselective angiogram shows that the wedged position has been achieved for the microcatheter (arrow). **E** Unsubtracted angiogram showing casting Onyx (circle). F: Right ICA angiogram shows that the volume of the BAVM nidus is reduced after EVT, and the draining vein is patent. Abbreviations: BAVM: brain arteriovenous malformation, CTA: computed tomography angiography, EVT: endovascular treatment, ICA: internal carotid artery, MCA: middle cerebral artery, M2-3: second and third segments of the MCA, R: right

**Fig. 7:**
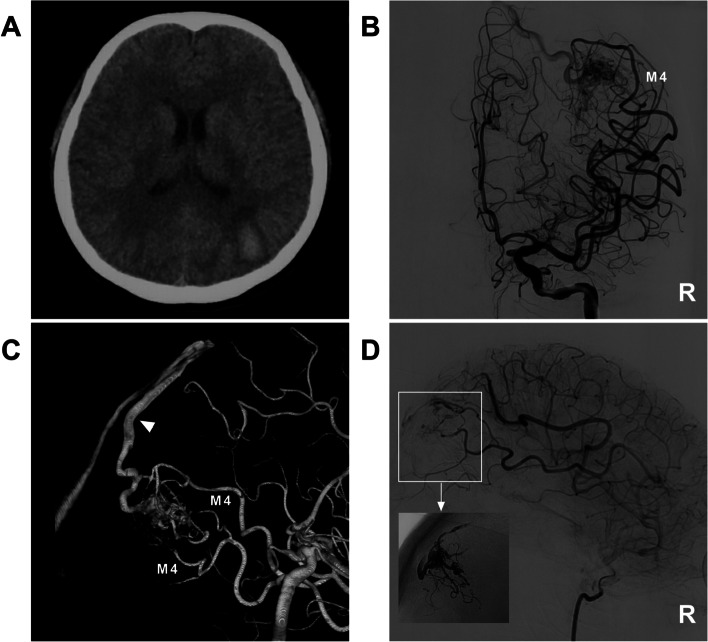
Curative EVT of a type IIIa BAVM. **A** CT showing hemorrhage in the left parietal lobe. **B** Anteroposterior angiogram of the right ICA showing that the BAVM was supplied only by the M4 segment. **C** Three-dimensional angiogram of the ICA showing that multiple branches of the M4 segment are involved as feeders, and only the superficial draining vein is observed (arrowhead). **D** Angiogram showing complete embolization of the BAVM (frame); the picture-in-picture shows the casting Onyx. Abbreviations: BAVM: brain arteriovenous malformation, CT: computed tomography, EVT: endovascular treatment, ICA: internal carotid artery, MCA: middle cerebral artery, M4: fourth segment of the MCA, R: right

**Fig. 8:**
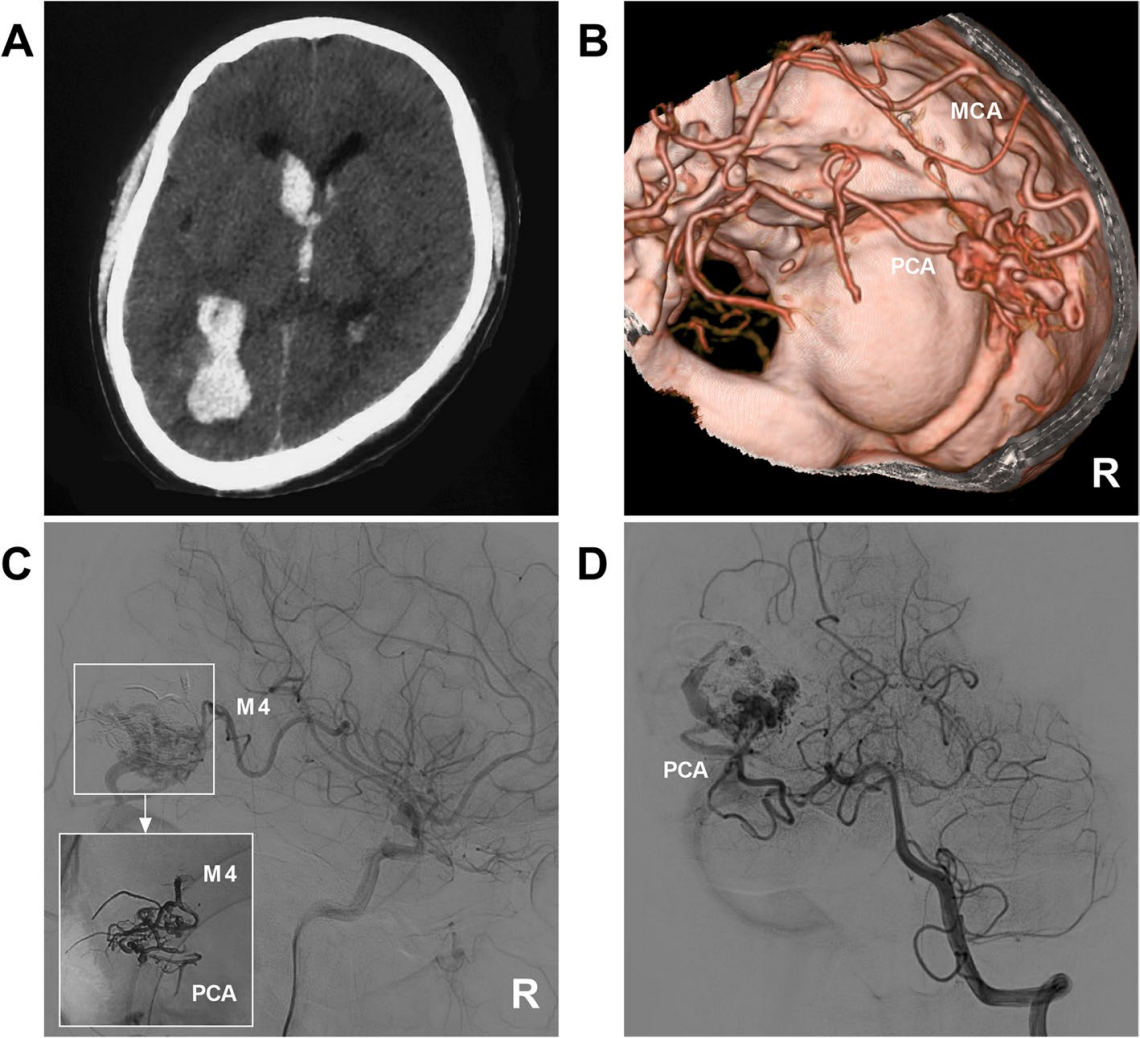
EVT of a type IIIb MCA-BAVM. **A** CT showed hemorrhage in the right parietal and occipital lobes extending into the ventricle. **B** CTA showing that the BAVM was supplied by the M4 segment of the MCA and PCA. **C** Right ICA angiogram showing that the BAVM was partially embolized via the MCA; the picture in the picture shows the casting Onyx. **D** Vertebral artery angiogram showing that the PCA still supplied the BAVM, but the volume of the BAVM nidus was reduced. Abbreviations: BAVM: brain arteriovenous malformation, CT: computed tomography, CTA: CT angiography, EVT: endovascular treatment, ICA: internal carotid artery, MCA: middle cerebral artery, M4: fourth segment of the MCA, PCA: posterior cerebral artery, R: right

Some cases of typical EVTs in this study are shown in Figs. 6, 7 and 8.

## Discussion

Many classification systems have been proposed for BAVMs, almost all of which are designed for surgical resection planning [18, 19]. For instance, the famous BAVM SM grading system, which was introduced by Spetzler and Martin in 1986, has proven to be useful in guiding surgical resection [20, 21]. The Lawton-Young Grading System improves the outcome prediction accuracy and is a feasible alternative to the SM system [22]. Recently, several grading systems for procedural risk in the EVT of BAVMs have been proposed, including the Buffalo, Puerto Rico, and BAVM embocure scoring systems [23–27]. However, these scoring systems are simply based on the number of feeders, AVM size, eloquence, and venous drainage. Therefore, Lv et al. chose factors of clinical, anatomical and angioarchitecture of the BAVM nidus and combined these factors to form a new Tsinghua grading system [28].

However, these grading systems did not aim at BAVMs in a certain location. In our previous reports, the classifications of BAVMs involving the ACA and PCA regions were proposed to evaluate the risk and EVT outcomes [2, 3]. Therefore, in our study, a similar classification should be proposed specifically for MCA-BAVMs to benefit EVT. When performing EVT for MCA-BAVMs, transarterial EVT remains the mainstream technique [29]. Therefore, classification of MCA-BAVMs based on the anatomical segment of the MCA is feasible because MCA‑BAVMs have a wide distribution along the MCA course.

MCA-BAVMs can be divided into four types, including types I, II, IIIa and IIIb. Because the distribution of the supplying areas of M1-4 segments varied, the incidence rate of MCA-BAVMs in each segment was different. For instance, in our study, Type III BAVMs were the most common, accounting for 77% of MCA-BAVMs, due to the large blood supply region of the M4 segment [30]. Compared with other grading systems, our classification system considers the unique characteristics of MCA-BAVMs along the MCA and is very individualized.

Due to the different anatomical characteristics of M1-4 segments of the MCA, such as branching, neighboring venous structures, and space, the angioarchitecture in different types of MCA-BAVMs is unique. In our study, no difference in age or sex was found among the types, which indicated that they did not affect the classification of MCA-BAVMs. In addition, in our study, the average age was 33.8 years, which is in accordance with the peak age of 30–34 years in Petridis et al.’s report on 6527 patients with BAVMs [31].

Under continually high pressure, BAVMs are not static, and their angioarchitectural features can develop with time. In adults, feeding artery aneurysms and ectasia of the draining vein are more often observed, suggesting that these particular features take time to develop [32].

Due to hemodynamic stress, the feeding artery and nidus may become dilated and thinned, and associated aneurysms can develop on the feeding artery or in the nidus of the BAVM. The incidence of associated aneurysms in all BAVMs was 20.2%, and the rate in supratentorial BAVMs was lower than that in infratentorial BAVMs [33]. Therefore, the rate of associated aneurysm in MCA-BAVMs should be less than 20.2%. In Stein et al.’s report of 409 patients with supratentorial BAVMs, 14.4% of patients suffered associated aneurysms [34]. In this study, the incidence of associated aneurysm was 14.8%, similar to that of Stein et al.’s report. Due to hemodynamic stress, remodeling of the primary draining vein is common, presenting with dilated or varicose morphology. In Pan et al.’s report of supratentorial BAVMs, the percentage of varicosities of the draining vein was 54.6% [35]. In this study, the morphology of the primary draining vein was varicose in 45.9% of BAVMs, similar to the above report.

Different types of MCA-BAVMs have unique imaging characteristics. Due to the limited space, type I BAVMs are smaller than type II and type IIIb BAVMs; in contrast, due to sufficient space, type IIIb BAVMs were larger than type I and type IIIa BAVMs. Because type I BAVMs have a deep location and type IIIb BAVMs have a large size, they can be close to the ventricle, and the deep vein is more often involved as the draining vein in them than other types of BAVMs. After rupture, IVH involvement was common. In addition, the remodeling of the primary draining vein in various types was different; the draining vein of Type IIIa BAVMs tended to retain normal morphology (*P* value > 0.05) (Table 5) because their only feeder is the MCA, the blood flow is low, and they are located on the hemisphere surface. The superficial veins often act as drainers; given their short course to the dural sinus, the low resistance reduces the probability of cortical vein remodeling [36]. However, in type IIIb BAVMs, multiple feeding arteries and a large nidus induced high-flow blood to remodel the primary draining vein.

For ruptured BAVMs and unruptured BAVMs with weak structures, intervention can be considered [10, 37]. EVT can be performed as a curative or useful adjunct treatment for MCA-BAVMs. Different types of BAVMs have different levels of difficulty and degrees of embolization during EVT. For Type I BAVMs, EVT poses a unique challenge due to the difficulty of LSA catheterization and the small diameter of the LSA [38, 39]. EVT is also difficult for Type II Sylvian BAVMs because the feeding arteries are often slim and disordered [16]. Therefore, type I and II BAVMs had low embolization degrees (*P* value > 0.05) (Table 5). However, Type IIIa BAVMs are easily treated with EVT and obtain a high embolization degree because of the simple angioarchitecture and ease of catheterization.

When performing EVT for BAVMs, the goal should not be an embolization degree, as the role of curative embolization is uncertain [40, 41]. In addition, in large BAVMs, the embolization of too large a volume in one stage may result in normal perfusion pressure breakthrough or occlusion of the draining vein, resulting in hemorrhagic complications [16, 17]. Therefore, in our study, the percentage of MCA-BAVMs with an embolization degree of > 2/3 nidus was only 36.3%. In addition, twenty risk-associated aneurysms were embolized. Therefore, the EVT results in this study were acceptable. In this study, 70.4% of patients with MCA-BAVMs suffered intracranial hemorrhage. In general, supratentorial hematoma can be tolerated. Therefore, EVT can be performed first, and then the hematoma can be managed with several alternatives, including conversative treatment and hematoma evacuation together without/with BAVM removal. In this study, 21.5% of patients experienced hematoma evacuation, and a good outcome with GOS scores of 4 and 5 was achieved in 97% of patients.

## Conclusion

The present study demonstrated that the new classification of MCA-BAVMs can be used to evaluate the imaging characteristics and EVT outcomes in different types. In addition, EVT may be a safe treatment modality for MCA‑BAVMs.

## Data Availability

The datasets used and/or analysed during the current study available from the corresponding author on reasonable request.
